# Chaperonin Contributes to Cold Hardiness of the Onion Maggot *Delia antiqua* through Repression of Depolymerization of Actin at Low Temperatures

**DOI:** 10.1371/journal.pone.0008277

**Published:** 2009-12-14

**Authors:** Takumi Kayukawa, Yukio Ishikawa

**Affiliations:** Laboratory of Applied Entomology, Graduate School of Agricultural and Life Sciences, The University of Tokyo, Tokyo, Japan; Institute of Evolutionary Biology (CSIC-UPF), Spain

## Abstract

Winter-diapause and cold-acclimated non-diapause pupae of the onion maggot, *Delia antiqua* (Diptera: Anthomyiidae), show strong cold hardiness. To obtain insights into the mechanisms involved in the enhancement of cold hardiness, we investigated the expression patterns of genes encoding subunits of chaperonin (CCT) and the morphology of actin, a substrate of CCT, at low temperatures. Quantitative real-time PCR analyses showed the mRNA levels of CCT subunits in pupal tissues to be highly correlated with the cold hardiness of the pupae. While actin in the Malpighian tubules of non-cold-hardy pupae showed extensive depolymerization after a cold treatment, actin in the same tissue of cold-hardy pupae was not depolymerized. Damage to cell membranes became apparent after the depolymerization of actin. Moreover, administration of Latrunculin B, an inhibitor of actin polymerization, to the larvae markedly decreased the cold hardiness of the pupae obtained. These findings suggest that CCT contributes to the cold hardiness of *D. antiqua* through the repression of depolymerization of actin at low temperatures.

## Introduction

Low temperatures endanger the life of animals and plants through chill injuries, even when well above freezing. One of the mechanisms that cause chill injuries in cells is the denaturation and subsequent aggregation of proteins [1], [2]. To avoid this, a wide range of organisms have acquired heat shock proteins (HSPs), which are classified into five major families based on molecular mass (small HSP, HSP60, HSP70, HSP90, and HSP100) [2]–[4]. Although HSPs usually work as molecular chaperones that fold and assemble newly synthesized proteins, they also prevent misfolding and aggregation of existent proteins, and convert proteins denatured under stressful conditions to their native conformations [2], [5]–[7].

Chaperonin (HSP60) in eukaryotes, often referred to as CCT (chaperonin containing t-complex polypeptide-1), is a hetero-oligomeric protein composed of 8 to 9 different subunit species of approximately 60 kDa [8]–[11]. Whereas the chaperonin in prokaryotes, GroEL, takes various substrates [12]–[15], until recently only actin and tubulin were known as substrates of CCT *in vivo*. However, numerous additional non-cytoskeletal proteins are now known to be substrates of CCT [10], [11], [16], [17]. GroEL expression is reported to be upregulated in response to various forms of stress including low temperature, and play important roles in resistance to stress [18]–[20]. In contrast, the roles of CCT under cold stress are not well understood, although production of CCT was recently shown to be induced by heat shock in human cells [21], by chemical stress (CdCl_2_) in the ciliate *Oxytricha granulifera* [22], and by cold shock in the yeast *Saccharomyces cerevisiae* [23].

**Figure 1:**
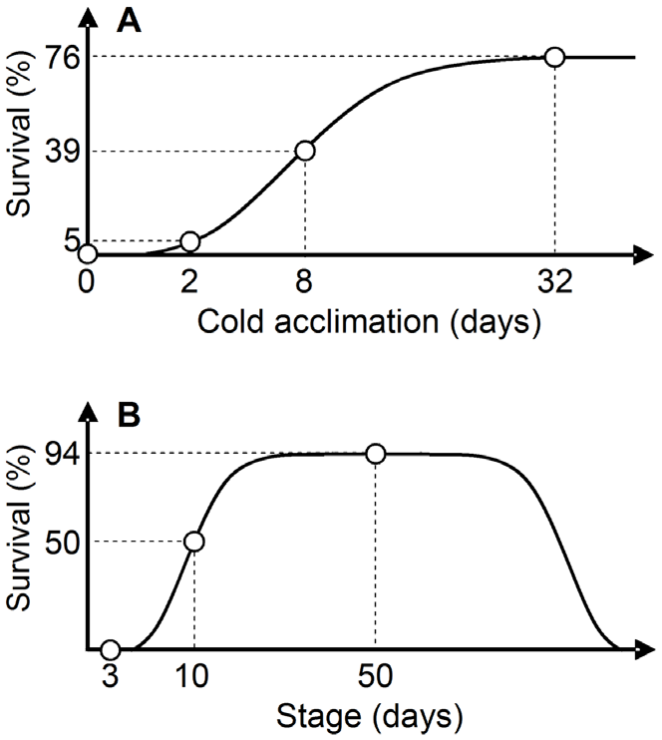
Schematic presentations of changes in cold hardiness of *Delia antiqua* pupae. Cold hardiness was assessed by percent survival after a 5-day treatment at −20°C [24]–[26]. (A) Non-diapause pupae acclimated at 5°C (CA). Larvae were reared at 23°C and 16L∶8D to prepare non-diapause pupae, and 5-day-old pupae were transferred to 5°C for a specified period before testing cold hardiness. (B) Winter-diapause pupae (WD). Larvae were reared at 15°C and 12L∶12D to prepare diapause pupae. Pupae were maintained under the same conditions as for rearing larvae.

We have long been interested in the strong cold hardiness of the diapause and cold-acclimated pupae of the onion maggot, *Delia antiqua*, a freeze intolerant insect. The percent survival of winter-diapause (WD) pupae after a 5-day exposure to −20°C exceeds 90% (Fig. 1) [24], [25]. Meanwhile, non-diapausing pupae also show a significantly increased survival rate when they are preexposed to 5°C (cold-acclimated non-diapause pupae, CA) (Fig. 1) [25], [26]. To explore the molecular mechanisms underlying the strong hardiness in *D. antiqua*, we sought for differentially expressed genes in the cold-acclimated and diapause pupae. Differential display (DD) analysis revealed the expression of a gene encoding TCP-1 (t-complex polypeptide-1), the α subunit of CCT, in *D. antiqua* (*DaTCP-1*) to be upregulated in some tissues of pupae that exhibit enhanced cold hardiness [25]. To clarify the relationship between the enhanced cold hardiness and upregulation of CCT, the present study investigated the expression patterns of genes encoding eight subunits of CCT and the changes in the higher structure of actin, the major substrate of CCT, at low temperatures.

## Results

### Cloning of All CCT Subunits and RACE

Fragments of all CCT subunits except the α subunit, which had already been obtained through a differential display analysis in the previous study [25], were obtained using degenerate PCR and RACE. The length of mRNA, predicted amino acid sequences and accession numbers for the Genbank® database are shown in Table S1. The eight CCT subunits of *D. antiqua* showed high amino acid identity (>70%) with corresponding CCT subunits of insect species, *Drosophila melanogaster*, *Anopheles gambiae*, *Tribolium castaneum*, *Apis mellifera*, and *Bombyx mori* (Table 1). Moreover, each subunit showed >57% identity with the corresponding subunit in human, yeast and rice, with the exception of the θ subunit, which showed somewhat lower identity (≥45%). The amino acid sequences of putative ATP-binding motifs in the N terminus of the eight subunits of *D. antiqua* showed a relatively high degree of identity (Fig. S1) [27], although the identity between subunits was only 23% to 44% when calculated based on the entire sequence (Fig. S1 and Table 2).

**Table 1:** Identity between the predicted amino acid sequence of each CCT subunit in *D. antiqua* and corresponding sequences in other insects, human, rice, and yeast a.

Species\Subunits	α	β	γ	δ	ε	ζ	η	θ
*D. melanogaster*	93%	86%	92%	90%	92%	85%	89%	84%
*A. gambiae*	90%	79%	81%	84%	83%	81%	85%	77%
*T. castaneum*	83%	83%	82%	78%	78%	81%	83%	74%
*A. mellifera*	82%	80%	79%	79%	77%	77%	77%	74%
*B. mori* b	-	80%	-	70%	-	81%	-	76%
*H. sapiens*	74%	73%	71%	66%	69%	75%	74%	69%
*O. sativa*	68%	68%	61%	59%	63%	63%	65%	53%
*S. cerevisiae*	64%	65%	57%	59%	58%	57%	62%	45%

a Abbreviations of the genus name and GenBank accession numbers of subunits are as follows. *D., Drosophila* (α: NP_524450, β: NP_572524, γ: NP_650572, δ: NP_609579, ε: NP_523707, ζ: NP_573066, η: NP_649835, θ: NP_610418); *A, Anopheles* (α: XP_313154, β: XP_318752, γ: XP_312164, δ: XP_310191, ε: XP_317219, ζ: XP_311767, η: XP_312160, θ: XP_314553); *T., Tribolium* (α: XP_969171, β: XP_970646, γ: XP_973604, δ: XP_973323, ε: XP_968355, ζ: XP_967748, η: XP_967459, θ: XP_975299); *A., Apis* (α: XP_392660, β: XP_393300, γ: XP_392814, δ: XP_623672, ε: XP_393315, ζ: XP_623697, η: XP_623090, θ: XP_623832); *B., Bombyx* (β: NP_001040109,δ: NP_001040107, ζ: NP_001040108, θ: NP_001073348); *H., Homo* (α: NP_110379, β: NP_006422, γ: NP_005989, δ: NP_006421, ε: NP_036205, ζ: NP_001753, η: NP_006420, θ: NP_006576); *O., Oryza* (α: BAH00555, β: AAT77033, γ: BAD54324, δ: BAD16520, ε: BAD53747, ζ: AAT93971, η: BAD45605, θ: AAS07232); *S., Saccharomyces* (α: NP_010498, β: NP_012124, γ: NP_012520, δ: NP_010138, ε: NP_012598, ζ: NP_010474, η: NP_012424, θ: NP_012526).

b The sequences of four CCT subunits in *B. mori* are not available in the GenBank database.

**Table 2:** Identity between the predicted amino acid sequences of CCT subunits in *D. antiqua*.

Subunits	α	β	γ	δ	ε	Ζ	η	θ
α	-	-	-	-	-	-	-	-
β	38%	-	-	-	-	-	-	-
γ	36%	32%	-	-	-	-	-	-
δ	31%	34%	35%	-	-	-	-	-
ε	40%	37%	30%	39%	-	-	-	-
ζ	28%	28%	33%	33%	34%	-	-	-
η	44%	38%	39%	43%	36%	34%	-	-
θ	30%	28%	31%	29%	29%	23%	31%	-

### Expression of Each CCT Subunit

**Figure 2:**
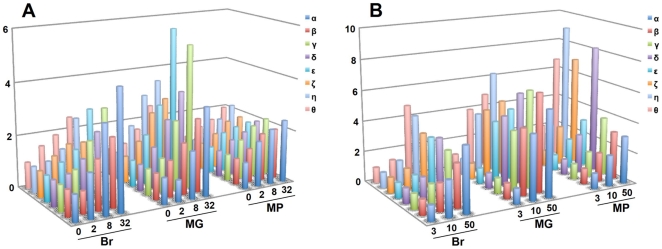
Quantitative real-time PCR analysis of *CCT subunit* mRNA levels. CCT subunit mRNA levels in Brain (Br), midgut (MG), and Malpighian tubules (MP) of cold-acclimated non-diapause pupae (A) and winter-diapause pupae (B). The levels of CCT subunit mRNA were normalized to those of the internal standard, 18S rRNA (three replicates). The horizontal and vertical axes represent the age of pupae, and fold-increase in the level of mRNA, respectively.

Changes in the mRNA levels of CCT subunits in association with cold acclimation and winter diapause were analyzed by Q-RT-PCR (Fig. 2). The expression of CCT subunits increased 2- to 6-fold in the brain, midgut and Malpighian tubules, as the period of cold acclimation was extended (Fig. 2A). In winter diapause pupae, the expression levels in all tissues examined were increased 2- to 9-fold (Fig. 2B). The mRNA levels of every CCT subunit in the three tissues of cold-acclimated and winter-diapause pupae increased in accordance with the increase in cold hardiness (Fig. 1, Fig. 2).

### Cell Membrane Injury and Structure of Actin Filaments at Low Temperature

**Figure 3:**
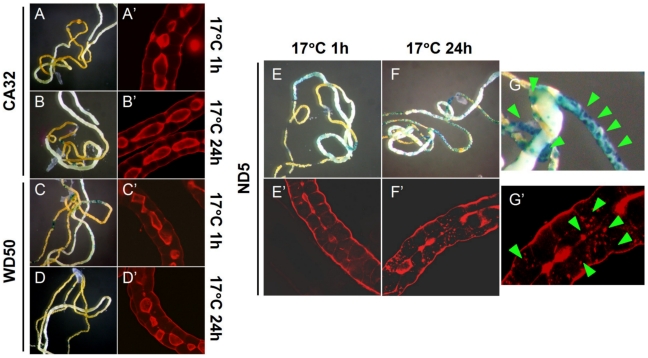
Effect of cold treatment on cell membrane and actin structure in Malpighian tubules. Cold-hardy (CA32 and WD50) and non-cold-hardy (ND5) pupae were treated at −20°C for 12days, and then maintained at 17°C for 1 h or 24 h. (A)–(G): Malpighian tubules were stained with Trypan Blue for detection of the damaged cell membrane. (A′)–(G′): Actin structures visualized with TRITC-phalloidin staining. (A), (A′), (B), and (B′): CA32 pupae. (C), (C′), (D), and (D′): WD50 pupae. (E), (E′), (F), (F′), (G), and (G′): ND5 pupae. (A), (A′), (C), (C′), (E), and (E′): after 1 h at 17°C. (B), (B′), (D), (D′), (F), (F′), (G), and (G′): after 24 h at 17°C. (G) and (G′) are enlargements of (F) and (F′), respectively. Arrowheads indicate disruptions of the cell membrane and actin filaments.

Cell membrane injury and the structure of actin were observed after staining with Trypan Blue and TRITC-phalloidin staining, respectively. Cell membranes in the Malpighian tubules of cold-hardy pupae (CA32 and WD50), maintained at 17°C for 1 or 24 h after exposure to −20°C for 12 days, were not disrupted (Fig. 3A–D). In contrast, those of ND5 were damaged by the incubation at 17°C for 24 h after the cold treatment, but not by the 1-h incubation (Fig. 3E–G). The distribution of actin filaments in the Malpighian tubules of *D. antiqua* was similar to that found in *Drosophila melanogaster* [28] (Fig. 3). Actin was found at the cell surface in Malpighian tubules, and was particularly concentrated at the apex (luminal surface) (Fig. 3A′–G′). Although no changes in the higher structure of actin were observed in the Malpighian tubules of CA32 and WD50 (Fig. 3A′–D′), depolymerization of actin was observed in ND5 pupae incubated at 17°C for 1 h after the cold treatment, and severe depolymerization of actin was observed after a 24-h incubation (Fig. 3E′–G′).

### Effect of the Inhibition of Repolymerization of Actin on Cold Hardiness

**Figure 4:**
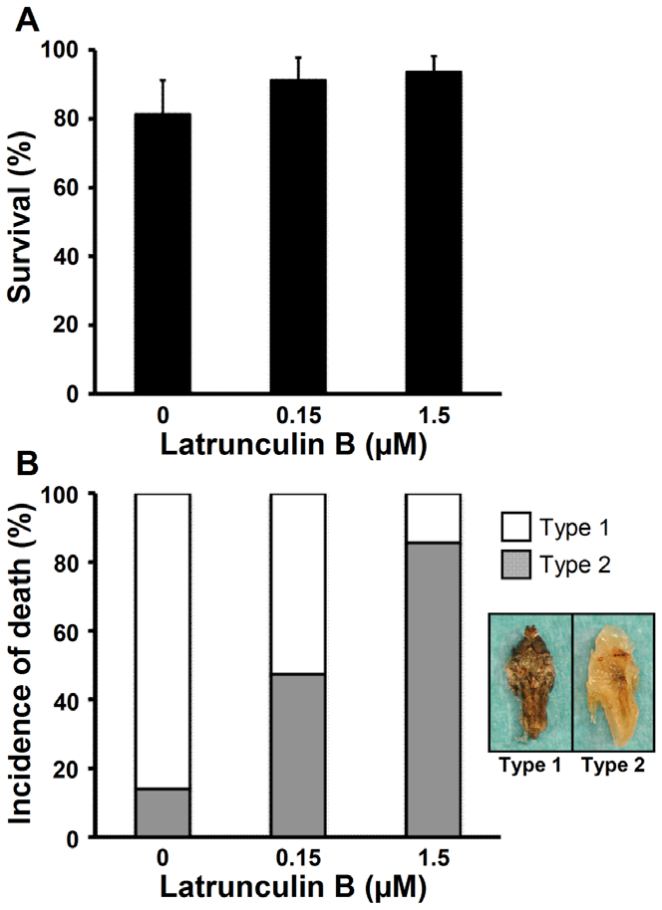
The effects of Latrunculin B treatment on cold hardiness. (A) Survival rates of ND5 pupae reared at 17°C and 16L∶8D after feeding on the diet containing Latrunculin B. (B) Incidence of the two types of death in ND5 pupae as affected by a 12-day treatment at −20°C. Type 1: the adult morphology appeared to have been attained [29]. Type 2: the pupal morphology was not changed from that of ND5 pupae [29].

The importance of the structure of actin to the cold hardiness of the cells was examined using an inhibitor of actin polymerization, Latrunculin B. Survival rates of ND5 pupae reared at 17°C and 16L∶8D after feeding on a diet containing 0, 0.15, or 1.5 µM Latrunculin B (Lat0, Lat0.15, and Lat1.5, respectively) were not significantly different, suggesting that the concentrations of Latrunculin B used in this study are not toxic to *D. antiqua* (Fig. 4A). ND5 pupae that eventually died after the exposure to −20°C for 5 days appeared to have died at two discrete developmental stages: after attaining the adult morphology (Type 1), and at a stage indistinguishable from ND5 in appearance (Type 2) (Fig. 4B) [29]. The difference in morphology between Type 1 and Type 2 represents the difference in the degree of cold injury: pupae exhibiting the Type-2 form suffered from severer damage than those exhibiting the Type-1 form [29]. Most (>80%) of the Lat0 (control) pupae exhibited Type-1 death, and a transition in the type of death, from Type 1 to Type 2, was observed as the concentration of Latrunculin B was increased (Fig. 4B). These results indicate that inhibition of the repolymerization of actin brought about rapid death of the pupae during the recovery from cold stress.

## Discussion

Our results present evidence that CCT contributes to the enhancement of cold hardiness in *D. antiqua* through repression of the depolymerization of actin at low temperatures. Recently, findings that suggest the importance of actin in cold hardiness have been made in several insects. In *Culex pipiens*, upregulation of two actin genes (*actin 1* and *actin 2*) and redistribution of actin were observed under cold stress and during diapause [30]. In *Sarcophaga crassipalpis*, the expression of *TCP-1*, which encodes the α subunit of CCT, was upregulated by the entering of diapause [4]. Moreover, in *Aphidius colemani*, the gene expression of an actin-depolymerizing factor was shown to be upregulated by low temperatures, suggesting that rearrangement of actin plays an important role in the survival of insects at low temperatures [31].

The changes in actin and tubulin, the major substrates of CCT [16], at low temperature have been investigated in several species of plants and animals. For example, actin filaments and microtubules of tobacco cells are depolymerized by low temperature, and are repolymerized during recovery from cold stress [32]. In winter wheat, *Triticum vulgare*, the disassembly and reorganization of microtubules occur during cold acclimation, and contribute to cold hardiness [33], [34]. Upadhya et al. proposed the following hypothesis on the mechanism of disassembly of actins based on experiments using a cell line of the rat liver [35], [36]. Low temperatures induce a decrease in the activity of Ca^2+^ATPase, and thereby a slow increase in the intracellular Ca^2+^ level, due to a unidirectional leak of Ca^2+^ from endoplasmic reticula (ER) because ER have higher Ca^2+^ levels than cytoplasm. The increase in Ca^2+^ levels brings about increased activity of calpain, which is known as a calcium-dependent intracellular protease that disassembles cytoskeletons including actin.

In *D. antiqua*, actin in the Malpighian tubules of cold-hardy pupae (cold-acclimated and diapause pupae) was not depolymerized by low temperatures, whereas actin in non-cold-hardy pupae showed extensive depolymerization after incubation at 17°C for 1 h or 24 h following the cold treatment (Fig. 3). Interestingly, damage to cell membranes was not apparent in 1 h at 17°C after the cold treatment, but became apparent after 24 h (Fig. 3). These results suggest that the disruption of cell membranes occurred after the depolymerization of actin. Thus, it was suggested that actin filaments are involved in the maintenance of cell membranes. The dense distribution of actin at the cell surface is consistent with this idea. Actually, inhibition of actin polymerization by Latrunculin B markedly decreased the survival rate of the pupae after cold treatment (Fig. 4).

The CCT subunits of *D. antiqua* showed 23–44% identity to each other (Table 2), the level of identity being similar to CCT subunits in mice (∼30%) [37]. In *Drosophila melanogaster*, CCT subunits are encoded by independent genes scattered on different chromosomes (FlyBase; http://flybase.bio.indiana.edu/). Interestingly, the gene expression of all CCT subunits in *D. antiqua* was synchronously increased in association with the increase in cold hardiness (Fig. 2). In mouse, the genes encoding CCT subunits respectively have heat shock elements (HSEs) in the 5′ non-coding region or in the first intron, and the transcription of CCT subunits is activated by the binding of heat shock transcription factors (HSFs) to HSEs [38]. Therefore, it is likely that the synchronous increase in the mRNA of CCT subunits at low temperature in *D. antiqua* is controlled by HSF. In *D. antiqua*, the expression of *HSP70* and *HSP90*, which also presumably have HSEs in their 5′ non-coding region, was upregulated by thermal stress and upon the entering of diapause [39], [40].

**Figure 5:**
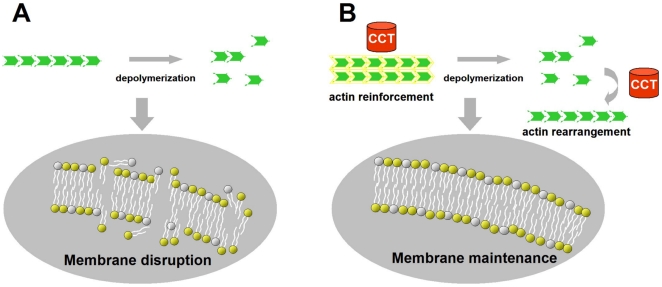
Suggested role of CCT in enhancing cold hardiness. Schematic representation of depolymerization of actin filaments at low temperature and the protection of actin filaments mediated by CCT. (A) non-cold-hardy pupae. (B) cold-hardy pupae.

Taken altogether, we consider the function of CCT and actin in the protection of cells from cold injury to be as follows (Fig. 5). In non-cold-hardy pupae of *D. antiqua*, low temperatures cause depolymerization of actin and subsequent disruption of cell membranes, although the involvement of Ca^2+^ and Ca^2+^ATPase in this process has not been verified in *D. antiqua* (Fig. 5A). In the cold-hardy pupae, the increased levels of CCT protein maintain the higher structure of actin through the suppression of depolymerization or rearrangement of actin monomers, which results in the maintenance of cell membranes and thereby the increase in cold hardiness (Fig. 5B).

## Materials and Methods

### Insects

Onion maggots were originally collected at Sapporo, Japan, and their offspring have been reared on an artificial diet for about 40 generations in the laboratory [41]. Adults were reared in fabric screen cages (30×30×30 cm) with a supply of water, sugar and yeast extract (Dried Yeast Extract D-3, Wako Pure Chemicals, Kyoto). The cages were maintained in an environmental chamber controlled at 23±1.0°C, under a 16 h light: 8 h dark photoperiod (16L: 8D) and 50–70% relative humidity. A plastic dish (8 cm in diameter ×5 cm) containing damp glass beads and several pieces of onion was placed in the cage as the oviposition substrate. Eggs were inoculated on a piece of artificial diet placed on moistened fine sand in a plastic container (13 cm in diameter ×8 cm). To obtain pupae with varied cold hardiness, larvae were reared under the conditions described below [24]. In the present article, the age of pupae is expressed by the days after pupariation, with the day of pupariation being day 0.

1) Cold-acclimated non-diapause pupae (CA). Larvae were reared at 20.0±0.2°C and 16L: 8D. After pupariation, the non-diapause pupae were maintained at 17.0°C±0.2°C and 16L: 8D for 5 days (ND5). ND5 pupae were then housed in a Styrofoam box and placed in an incubator adjusted to 5.0±0.2°C for 0, 2, 8 or 32 days (CA0, CA2, CA8 or CA32). Because the lower threshold temperature for pupal development in *D. antiqua* is 5.7°C [42], no development was considered to have occurred during acclimation. Hence, all cold-acclimated pupae were regarded as ND5, irrespective of the duration of acclimation.

2) Winter-diapause pupae (WD). Larvae were reared at 15.0±0.2°C and 12L: 12D. Under these conditions, >95% of pupae entered winter diapause. Pupae were maintained under the same conditions as for rearing larvae for 3, 10 or 50 days (WD3, WD10 or WD50). WD3 pupae were in a pre-diapause state, while WD10 and WD50 pupae were in diapause.

### Dissection

The thin outermost shell of the puparia (puparial case) was removed using fine forceps, and the pupa was dissected in cold phosphate-buffered saline (PBS, 137 mM NaCl, 8 mM Na_2_HPO_4_, 2.7 mM KCl, and 1.5 mM KH_2_PO_4_, pH 7.4). The brain, midgut, and Malpighian tubules were isolated from 25 to 30 animals and snap frozen in liquid nitrogen. These tissues were chosen because they could be easily isolated and occupied a large portion of the pupal body.

### Degenerate PCR

mRNA was extracted from the brains of WD50 pupae using a MicroPoly (A) Purist™ kit (Ambion). cDNA was then synthesized using a TaKaRa RNA PCR Kit (AMV), Ver.3.0 (TaKaRa). Degenerate PCRs for amplifying gene fragments encoding seven subunits (β,γ, δ, ε, ζ, η, and θ) of CCT were conducted using the primers listed in Table S2. Thermal cycling conditions were 94°C for 3 minutes, 35 cycles of 98°C for 10 s, 48°C for 30 s and 72°C for 2 min, and 72°C for 5 min. The PCR products were separated on a 2% agarose gel, and stained with SYBR™ Green I (Molecular Probes). The fragment of expected size was extracted from the agarose gel, and cloned using pGEM®-T easy vector system I (Promega). The inserts were sequenced using a BigDye Terminator v1.1 Cycle Sequencing Kit (Applied Biosystems) on an ABI PRISM 377HN Sequencer (Applied Biosystems).

### Cloning of cDNA Fragments by RACE

The 5′ and 3′ ends of the cDNA of CCT subunits were obtained by a modified RACE method using a FirstChoice® RLM RACE Kit (Ambion). The first-strand cDNA was synthesized from mRNA extracted from the brains of WD50 pupae. The first PCR in the 5′ RACE (3′ RACE) was performed using the 5′ RACE (3′ RACE) outer primer supplied in the kit (Ambion) and primers specific for the respective subunit (Table S2). The nested PCRs were performed using the 5′ RACE (3′ RACE) inner primer and primers specific for the respective subunit (Table S2). The PCR products obtained by 5′ and 3′ RACE were subcloned using pGEM®-T Easy Vector System I (Promega) and sequenced.

### Quantitative Real-Time PCR (Q-RT-PCR)

Total RNA was extracted from the brain, midgut, and Malpighian tubules using an RNeasy mini kit and RNase-free DNase I (Qiagen). The cDNAs synthesized by using a SYBR® RT-PCR Kit (Takara) were diluted to 1/50 for the detection of CCT subunits and 1/1000 for the 18S rRNA gene (control). The primers used for Q-RT-PCR for CCT subunits are shown in Table S2, and the primers for the 18S rRNA gene were 5′-TTAAGCCATGCATGTCTAAGTAC-3′ and 5′-TCTCAGGCTCCCTCTCCGGAATCG-3′. The transcripts of CCT subunits were quantified using an ABI PRISM 7700 thermal cycler (Applied Biosystems). The Q-RT-PCR was carried out in a 20-µl reaction volume containing 1 µl of template cDNA, SYBR Premix Ex Taq, ROX Reference Dye (Takara) and 0.2 µM each of the primers. Shuttle PCR conditions were 95°C for 10 s followed by 40 cycles of 95°C for 5 s and 60°C for 30 s. After Q-RT-PCR, the absence of by-products was confirmed by a melting curve analysis and agarose gel electrophoresis of the PCR products. The relative molar amounts of CCT subunits and 18S rRNA transcripts were calculated based on a crossing point analysis, using standard curves generated from a cDNA standard. mRNA levels of CCT subunits were normalized with those of 18S rRNA in the same samples quantified in the same manner (three replicates). The relative mRNA levels of CCT subunits in each tissue of CA0 and WD4 pupae were set as 1.

### Cold Treatment of Pupae

To avoid the inoculative freezing of pupae, the surface of the pupae was dried at the rearing temperature, and moist filter paper in the Petri dish was replaced with dry filter paper before the exposure to −20°C. The non-diapause, cold-acclimated and winter-diapause pupae were exposed to −20±0.2°C and 0L∶24D for 12 days.

### Staining of Damaged Cells and Actin

After exposure to −20°C for a defined period, the pupae were maintained at 17±0.2°C and 16L∶8D for 1 or 24 h. Then the pupa was dissected in PBS as described above. The Malpighian tubules were isolated within 2 min, and immersed in Trypan Blue solution (3 mg/ml PBS) for 3 min at room temperature to stain the cells with a disrupted membrane. The tissues were rinsed in PBS three times, and immediately inspected with a stereomicroscope.

For staining actin, Malpighian tubules were fixed for 10 min in 4% formaldehyde in PBS, washed twice in PBS containing 0.1% Triton-X-100 (PBST), and stained overnight at 4°C with TRITC-phalloidin (Sigma) in PBST containing 3% bovine serum albumin [43]. The tissues were analyzed using a confocal laser scanning microscope (Zeiss LSM5 Pascal).

### Administration of Latrunculin B

Ten-day-old larvae reared under the non-diapause conditions were fed an artificial diet containing 0, 0.15, or 1.5 µM of Latrunculin B, an inhibitor of actin polymerization, until they pupariated. ND5 pupae in Petri dishes (50 pupae/dish) were exposed to −20±0.2°C for 5 days, and then maintained at 17±0.2°C and 16L∶8D. The survival rate and the morphology of pupae were examined 25 days after the exposure to −20°C (3 replicates).

## Supporting Information

Figure S1 Alignment of part of predicted amino acid sequences of CCT subunits of *D. antiqua*. The underlined amino acids represent the presumed ATP-binding motifs in the N terminus.
(1.88 MB PPT)

Table S1 The length and accession number of each CCT subunit.
(0.05 MB DOC)

Table S2 PCR primers used in this study.
(0.06 MB DOC)
