# Supplementary figures and images for: Chaperonin Contributes to Cold Hardiness of the Onion Maggot Delia antiqua through Repression of Depolymerization of Actin at Low Temperatures
Source: PLoS One. 2009 Dec 14;4(12):e8277. doi: 10.1371/journal.pone.0008277 (PMC2788269; doi:10.1371/journal.pone.0008277)

## Slide 1
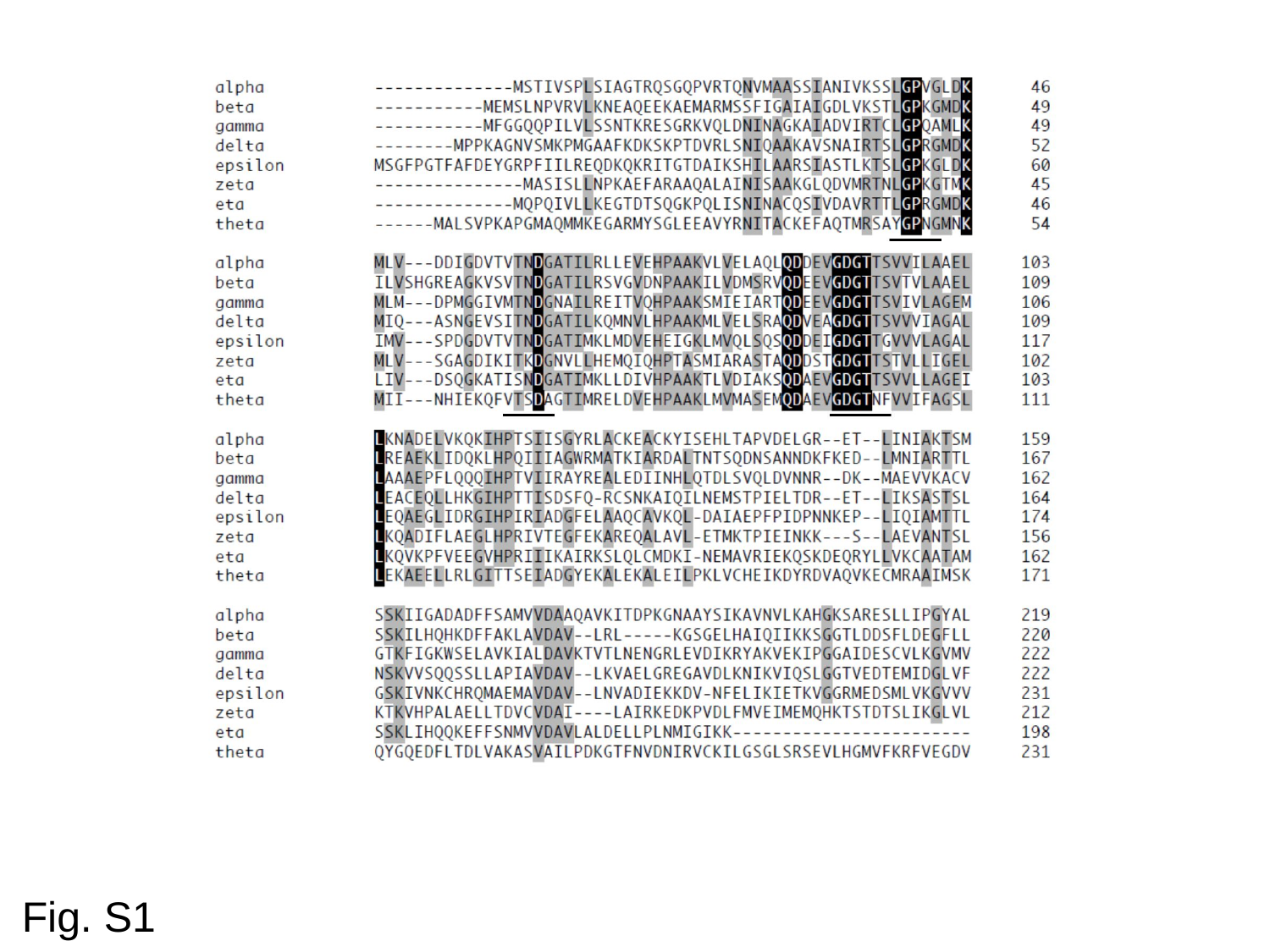

Fig. S1

Supplement: Figure S1 — Alignment of part of predicted amino acid sequences of CCT subunits of D. antiqua. The underlined amino acids represent the presumed ATP-binding motifs in the N terminus. (1.88 MB PPT) [file pone.0008277.s001.ppt]
