# Supplementary material for: Chaperonin Contributes to Cold Hardiness of the Onion Maggot Delia antiqua through Repression of Depolymerization of Actin at Low Temperatures
Source: PLoS One. 2009 Dec 14;4(12):e8277. doi: 10.1371/journal.pone.0008277 (PMC2788269; doi:10.1371/journal.pone.0008277)
Supplement: Table S1 — The length and accession number of each CCT subunit. (0.05 MB DOC) [file pone.0008277.s002.doc]

Table S1. The length and accession number of each CCT subunit.

| CCT subunit | length | | accession no |
| --- | --- | --- | --- |
|  | mRNA | amino acids |  |
| β | 964 bp | 300 a.a. | AB430832 |
| γ | 845 bp | 249 a.a. | AB430833 |
| δ | 1382 bp | 415 a.a. | AB430834 |
| ε | 944 bp | 285 a.a. | AB430835 |
| ζ | 1055 bp | 316 a.a. | AB430836 |
| η | 742 bp | 198 a.a. | AB430837 |
| θ | 972 bp | 301 a.a. | AB430838 |
