# Supplementary material for: Chaperonin Contributes to Cold Hardiness of the Onion Maggot Delia antiqua through Repression of Depolymerization of Actin at Low Temperatures
Source: PLoS One. 2009 Dec 14;4(12):e8277. doi: 10.1371/journal.pone.0008277 (PMC2788269; doi:10.1371/journal.pone.0008277)
Supplement: Table S2 — PCR primers used in this study. (0.06 MB DOC) [file pone.0008277.s003.doc]

Table S2. PCR primers used in this study.

| name | Nucleotide sequence (5’ to 3’) |
| --- | --- |
| alpha subunit |  |
| Q-RT-PCR FW | CGGCGGTGTTGATGATTTGT |
| Q-RT-PCR RV | GCAGCACCAGTTGCCTTAGC |
| beta subunit |  |
| degenerate FW | CCNAARGGNATGGAYAA |
| degenerate RV | AARTCNGCRTGYTCRAT |
| RACE FW1 | GGTGCCACTATTTTGCGTTCTGT |
| RACE FW2 | CCCGAGATGCTTTAACCAACACTT |
| RACE RV1 | GTGGTGCGGGCAATATTCATTA |
| RACE RV2 | GCCCACTTCTTCATCTTGGACAC |
| Q-RT-PCR FW | GGTGCCACTATTTTGCGTTCTGT |
| Q-RT-PCR RV | GCCCACTTCTTCATCTTGGACAC |
| gamma subunit |  |
| degenerate FW | AAYGAYGGNAAYGCNAT |
| degenerate RV | TCNCCYTTYTTRTAYTC |
| RACE FW1 | CCGATTTAAGCGTTCAGTTGGA |
| RACE FW2 | GGTGCCATTGATGAATCCTGTG |
| RACE RV1 | GAACAATCGAGCAAGACAATACGG |
| RACE RV2 | TACCGCCAATTCCGACCATT |
| Q-RT-PCR FW | GGTGCCATTGATGAATCCTGTG |
| Q-RT-PCR RV | GAACAATCGAGCAAGACAATACGG |
| delta subunit |  |
| degenerate FW | GGNATGGAYAARATGAT |
| degenerate RV | ATYTCNGGNGCNCCRCC |
| RACE FW1 | CGAAACCCACTGATGTTCGTCTA |
| RACE FW2 | GGACTCTTTCCAACGTTGTTCCA |
| RACE RV1 | GCCAACAAACTGCTCTGTTGAGA |
| RACE RV2 | ATACCACGGGGTCCTAAACTGGT |
| Q-RT-PCR FW | CGAAACCCACTGATGTTCGTCTA |
| Q-RT-PCR RV | ATACCACGGGGTCCTAAACTGGT |
| epsilon subunit |  |
| degenerate FW | AAYGAYGGNGCNACCAT |
| degenerate RV | TTATGCTTNGTYTTNGG |
| RACE FW1 | AATTGGTGATGGTACCACTGGTGT |
| RACE FW2 | ACCTTTTCCCATTGATCCCAAC |
| RACE RV1 | ACACGGCATCAACAGCCATC |
| RACE RV2 | AATGCCACGATCGATGAGTCC |
| Q-RT-PCR FW | ACCTTTTCCCATTGATCCCAAC |
| Q-RT-PCR RV | ACACGGCATCAACAGCCATC |
| zeta subunit |  |
| degenerate FW | AACATCARYGCYTGYCA |
| degenerate RV | ATYTCRAASCCNGCRTA |
| RACE FW1 | TGCCAAGGGTTTGCAAGATG |
| RACE FW2 | CGCGCTAGTACCGCTCAAGA |
| RACE RV1 | TTCGGTAACAATGCGTGGATG |
| RACE RV2 | TCACCAGCACCAGATACCAACA |
| Q-RT-PCR FW | TGCCAAGGGTTTGCAAGATG |
| Q-RT-PCR RV | TCACCAGCACCAGATACCAACA |
| eta subunit |  |
| degenerate FW | AACATCARYGCYTGYCA |
| degenerate RV | ATYTCRAASCCNGCRTA |
| RACE FW1 | GTCCTCGTGGCATGGACAAA |
| RACE FW2 | GCCACAGCCATGTCTTCCAA |
| RACE RV1 | CAGGGCGAGAACAGCATCAA |
| RACE RV2 | GGCGATGTCGACCAATGTCT |
| Q-RT-PCR FW | GCCACAGCCATGTCTTCCAA |
| Q-RT-PCR RV | CAGGGCGAGAACAGCATCAA |
| theta subunit |  |
| degenerate FW | GGCATGGTNTTYAARCG |
| degenerate RV | TGNAGAGCCATRTCKCC |
| RACE FW1 | ATCTGTACCCAAAGCACCTGGAA |
| RACE FW2 | GTGAAATGCAAGACGCTGAAGTG |
| RACE RV1 | TTCCGAGGTAGTAATGCCCAAAC |
| RACE RV2 | GATACACGGCCTCTTCCAAACC |
| Q-RT-PCR FW | ATCTGTACCCAAAGCACCTGGAA |
| Q-RT-PCR RV | GATACACGGCCTCTTCCAAACC |
